# Cadmium Contamination in Asian Rice (*Oryza sativa* L.): Mechanistic Insights from Soil Sources to Grain Accumulation and Mitigation Strategies

**DOI:** 10.3390/plants14182844

**Published:** 2025-09-12

**Authors:** Jing Wang, Bian Wu, Lei Zhou, Kai Liu, Aiqing You, Wenjun Zha

**Affiliations:** 1 Key Laboratory of Crop Molecular Breeding, Ministry of Agriculture and Rural Affairs, Hubei Key Laboratory of Food Crop Germplasm and Genetic Improvement, Food Crops Institute, Hubei Academy of Agricultural Sciences, Wuhan 430064, China; jing2567@webmail.hzau.edu.cn (J.W.); wubian@hbaas.com (B.W.); zhoulei@hbaas.com (L.Z.); liukai11153@hbaas.com (K.L.); 2 Hubei Hongshan Laboratory, Wuhan 430070, China

**Keywords:** rice, cadmium contamination, transport mechanisms, cadmium pollution control

## Abstract

Cadmium (Cd) pollution in rice crops is a global environmental challenge, endangering food security and sustainable agricultural development. Cd ions are highly dynamic and toxic and can easily accumulate in rice grains, resulting in adverse consequences on human health and ecological safety. With accelerated industrialization and abundant agricultural activities, Cd enters paddy soils through multiple pathways, leading to increasingly complex processes of migration and transformation of Cd in the soil–rice ecosystem. Although recent studies have substantially advanced our comprehension of the pathways promoting the uptake, transport, and accumulation of Cd in rice, this information is scattered and lacks systematic integration, leading to an incomplete understanding of the entire contamination process. This review adopts a rigorous perspective spanning from soil input to grain accumulation and comprehensively summarizes the absorption pathways, translocation mechanisms, and remediation strategies for Cd pollution in rice. The effects of phytotoxicity induced by Cd on rice growth are thoroughly analyzed, and recent advances in various mitigation strategies are highlighted, including agronomic management, cultivar improvement, bioremediation, and signal regulation. By integrating the findings of latest research, this review (i) proposes a mechanistic network of Cd contamination occurrence and control in rice; (ii) elucidates critical regulatory nodes; and (iii) offers a theoretical framework for growing rice cultivars with a low Cd content, remediating Cd-contaminated farmlands, and ensuring food safety.

## 1. Introduction

Cadmium (Cd) is a toxic element and an environmental risk factor with high biotoxicity, commonly referred to as a “heavy metal,” although this term lacks a universally accepted definition. In this paper, “heavy metal” is used in the broad sense commonly adopted in the environmental science literature. Although the natural background levels of Cd are relatively low, rampant human activities—such as excessive industrialization, unregulated urban expansion, and overuse of agricultural inputs—have resulted in substantial Cd emissions into soil, water, and atmosphere, thereby causing gradual buildup of Cd in agricultural lands [1,2]. A concerning issue related to soil Cd contamination is its high toxicity and non-biodegradability; these properties contribute to its prolonged presence in the environment. This persistence of Cd not only disrupts ecosystem functionality but also endangers food security and human health [3]. Based on historical records, Cd contamination primarily resulted in Itai-Itai disease in Toyama Prefecture, Japan, during the 20th century [4]. Furthermore, epidemiological evidence has established strong correlations between Cd exposure and various cancers [5], respiratory illnesses [6], hepatic disorders [7], and renal dysfunctions [8]. Hence, it is imperative to comprehensively assess the pathways involved in the uptake, transportation, and accumulation of Cd in rice; elucidate the associated regulatory networks; and develop practically feasible potential intervention strategies to ensure sustained agricultural productivity and mitigate environmental pollution.

Against this background, rice (*Oryza sativa* L.), as one of the most important staple crops worldwide and the primary food source for more than half of the global population [9], plays a critical role in ensuring food safety, which is directly linked to human health and social stability. China is a leading rice producer, with high rice consumption; in particular, southern China, where rice is traditionally consumed as the dominant staple food, is a major rice-producing region. According to the 2024 data from the National Bureau of Statistics, China has a rice cultivation area of 290,100 km^2^, with a total annual production of 207.535 million tons, representing 31.8% of the nation’s total grain output [10]. In recent years, following increasing migration and evolving dietary patterns, there has been an increasingly high domestic demand for rice and its derivative food products, rendering rice a staple food widely consumed by both urban and rural Chinese populations [11,12].

Although rice is one of the most important staple crops in China, its efficient uptake and accumulation of Cd poses a serious threat to food security. According to the standards set by the Food and Agriculture Organization (FAO), Cd concentrations exceeding 0.2 mg/kg in rice could pose a risk to human health [13]. In fact, studies have shown that rice is one of the crops with the highest Cd accumulation [14,15], especially in rice grown in Cd-contaminated soils, where the root contains the highest levels of Cd, followed by the stem and leaves, with the lowest concentration in the grains. Although the cadmium content in the grains typically ranges from 0.1–0.3 mg/kg, in high-accumulating varieties or heavily contaminated environments, the cadmium concentration can exceed 0.4 mg/kg [15,16]. This situation results in a daily Cd intake of 20–40 μg/d for Chinese populations whose main staple is rice, posing potential health risks [17]. According to statistical data, approximately 33.2–33.5% of agricultural land in China is affected by cadmium pollution, with cadmium concentrations in the soil ranging from 0.01–1 mg/kg, and in some severely polluted areas, it can even reach 5.5 mg/kg [18,19]. Hence, it is vital to mitigate the issue of Cd contamination in China’s rice production areas. As Asia is the primary region for rice production and consumption, and Cd contamination in paddy fields is particularly severe there, this review focuses on studies from Asia. We also note that similar issues may occur in other rice-producing regions.

To date, several studies have examined various aspects of rice contamination with Cd, such as Cd speciation and transformation in soil [20], the effect of soil physicochemical properties on Cd bioavailability [21,22], absorption- and transportation-related pathways of Cd in rice [23,24,25], and the toxic effects of Cd on the development and growth of rice [26,27,28]. Concurrently, multiple studies have focused on screening rice cultivars with a low ability to accumulate Cd [29,30], optimizing fertilization and irrigation practices [31,32,33], and designing diverse soil remediation and agronomic management strategies [34,35,36,37], leading to notable progress in preventing and monitoring Cd contamination in rice. However, the information derived from these studies is scattered, and a comprehensive synthesis of the gained knowledge is lacking, resulting in an incomplete framework for understanding the mechanisms and control of Cd pollution. Therefore, in the present review, we systematically organize the findings of current research spanning the entire process of migration of Cd from the environment to rice grains, with an aim to provide a theoretical foundation and practical reference for elucidating Cd accumulation mechanisms and developing pollution mitigation strategies. The review integrates existing findings from a holistic perspective, with a focus on the following key aspects:

1. An overview of the primary sources of Cd contamination in agricultural ecosystems;
2. A summary of the effects of Cd contamination on rice, including its migration, uptake, and accumulation within the soil–rice system;
3. A synopsis of the prevailing remediation technologies and management strategies for mitigating Cd contamination.

This review aims to present the current status and future directions of Cd pollution research, providing a theoretical framework and practical guidelines for mitigating Cd contamination in rice cultivation, improving farmland ecological health, and ensuring food safety.

## 2. Cd Contamination in Soil and Its Sources

Under natural conditions, Cd primarily enters soil through weathering of rocks [38,39], volcanic eruptions [40], and release from ores [41]. These natural sources contribute to a relatively low amount of Cd in soil, thus posing limited ecological risk. However, the rapid growth of industrialization and urbanization as well as unregulated agricultural production have caused substantial environmental emission of Cd through mining and smelting, industrial discharge, agricultural inputs, and daily human activities. Consequently, there has been progressive Cd accumulation in agricultural soils, making anthropogenic sources the predominant factor of Cd pollution, thereby severely threatening ecosystem functions and human health [42,43,44,45].

Although human activities primarily contribute to Cd contamination in paddy soils, the dominant sources vary regionally, with no consensus on their proportional contributions. According to existing studies, mining and smelting [19,45], electronic equipment manufacturing [46], phosphate fertilizer application [47,48], and sewage irrigation [49,50,51] are the major pathways through which Cd enters the soil. The relative impact of each of these pathways is influenced by the region’s industrial structure, agricultural practices, and environmental regulations. For instance, in China, phosphate fertilizer use led to total Cd input of 10.52 tons in 2016, with 83.31% of this input being contributed by diammonium phosphate and monoammonium phosphate [52]. Additionally, particulate matter released during mining and smelting processes deposits Cd into soils through atmospheric pathways, further exacerbating soil Cd accumulation [53,54]. Because of high water demand for plant growth, some mining regions often utilize low-cost mining wastewater for irrigating rice crops, which elevates Cd concentrations in downstream paddy fields. Considerable variations in industrialization levels, urbanization, agricultural development, and soil properties across countries have resulted in marked regional disparities in Cd pollution levels in soils used for rice cultivation. For example, the average Cd level is 0.01–5.50 mg/kg (median value: 0.23 mg/kg) in Chinese paddy soils [18]; in contrast, soils in Pakistan and Bangladesh exhibit high Cd levels of 1.6–2.3 and 0.83–4.08 mg/kg, respectively, indicating a severe pollution risk [55,56].

## 3. Toxic Effects of Cd in Rice

Following its absorption in rice plants, Cd accumulates in tissues and exerts substantial toxicity that disrupts cellular structures and hinders multiple metabolic processes, thereby impairing normal physiological functions and developmental processes. Cd stress markedly inhibits rice plant growth, which decreases root and shoot lengths and reduces biomass buildup; moreover, these inhibitory effects increase following elevated Cd concentrations [27,57,58,59]. Moreover, Cd impairs the photosynthesis process through multiple mechanisms. First, Cd decreases chlorophyll content, disrupts chloroplast ultrastructure, and lowers photosystem II (PSII) activity and electron transport chain efficiency, leading to reduced light energy absorption and conversion and a lower net photosynthetic rate [60,61,62,63]. Second, Cd inhibits enzymes with a critical function in photosynthesis, such as ribulose-1,5-bisphosphate carboxylase/oxygenase (Rubisco), thereby hindering carbon assimilation and diminishing the synthesis of photosynthetic products [64,65]. Moreover, Cd stress can lead to various physiological disorders, including damage to the photosynthetic apparatus and membrane structures, as well as disruption of carbon metabolism and energy supply [66,67,68]. At the stomata level, Cd indirectly induces stomatal closure, limiting CO_2_ uptake and further constraining photosynthesis [27]. Although certain Cd-tolerant rice cultivars enhance their stress adaptation by upregulating photosynthesis-related proteins and antioxidant enzymes, the complete restoration of photosynthetic efficiency is difficult. Notably, exogenous application of nanomaterials such as silicon, zinc, and selenium can partially alleviate Cd-induced photosynthetic inhibition, thereby improving the growth of rice plants and their photosynthetic performance [69,70,71]. However, the use of certain nanomaterials may pose potential risks to ecosystems and human health.

**Figure 1:**
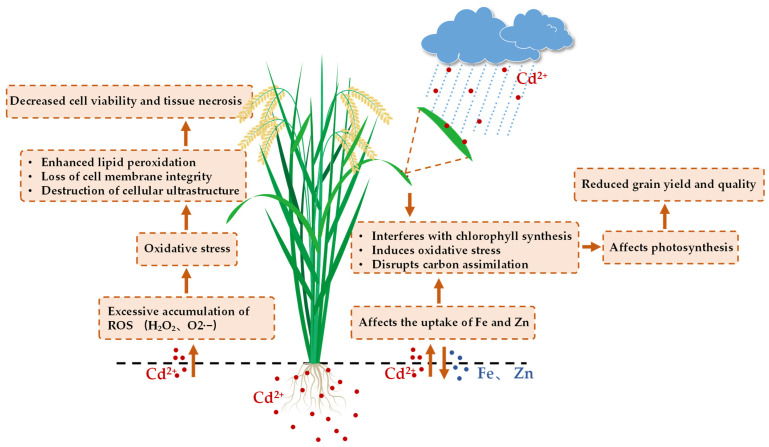
Schematic Representation of Toxicological Responses in Rice to Cadmium Exposure.

Cd stress induces excessive ROS accumulation, including hydrogen peroxide (H_2_O_2_) and superoxide anions (O_2_^−^), in rice tissues, causing severe oxidative stress in cells and subsequent cellular damage [67,68,72]. This oxidative stress manifests as enhanced lipid peroxidation, elevated malondialdehyde level, impaired membrane integrity, and disrupted cellular ultrastructure, ultimately causing reduced cell viability and tissue necrosis. Moreover, Cd directly damages the activity and molecular structure of antioxidant enzymes such as peroxidase (POD), superoxide dismutase (SOD), and catalase, thereby weakening their ROS scavenging capacity and exacerbating oxidative injury [73]. To reduce oxidative stress, rice activates its antioxidant defense system by upregulating enzymatic activities and accumulating non-enzymatic antioxidant compounds, including flavonoids, ascorbic acid, and glutathione. Cd stress also modulates the expression of genes associated with cell wall repair, glutathione metabolism, and hormone signaling pathways to enhance tolerance [74,75,76,77]. Exogenous application of silicon, citric acid, and microbial extracellular polymers can minimize oxidative damage by reinforcing the antioxidant system and reducing plant Cd absorption and transport [68,72,78]. These toxic effects are conceptually summarized in Figure 1.

## 4. Cd Absorption and Transportation Pathways in Rice

**Figure 2:**
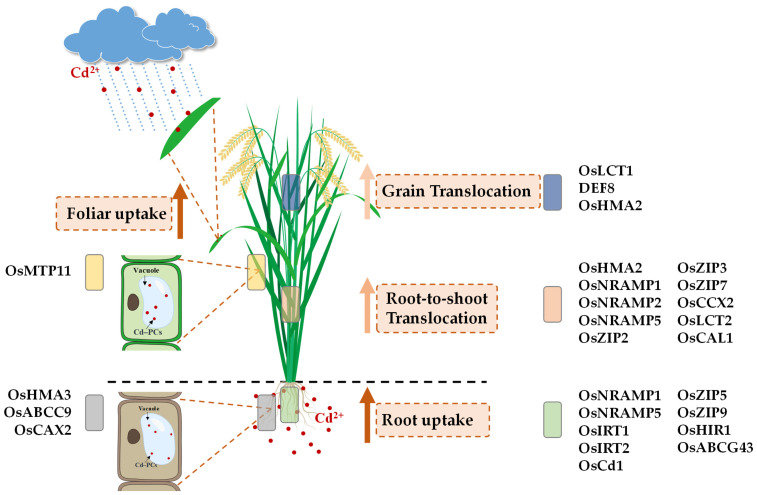
Overview of Cadmium Transport and Transporter Distribution in Rice.

In rice, Cd absorption primarily occurs through the root and foliar pathways. Following its entry, the transportation and accumulation of Cd involves four major processes: (1) translocation from roots to aerial tissues; (2) xylem-mediated transport to stems and leaves, leading to accumulation in vegetative organs; (3) direct foliar uptake through stomatal entry or surface adsorption; and (4) subsequent phloem-mediated redistribution and enrichment in grains. These coordinated pathways are schematically illustrated in Figure 2, while the key transport proteins involved in each pathway are summarized in Table 1. Together, these coordinated pathways regulate the spatial distribution and accumulation patterns of Cd in rice, thereby critically influencing grain safety.

### 4.1. Root Uptake

Roots are the primary plant organ controlling mineral nutrient uptake. Cd, a nonessential and toxic heavy metal, primarily enters rice roots through (1) the apoplastic pathway: Cd undergoes passive diffusion along intercellular spaces and cell walls, driven by the external concentration gradient and independent of energy input. However, upon reaching the root endodermis, its apoplastic transport is obstructed by the Casparian strip, preventing further entry into the stele through the apoplastic route. (2) The symplastic pathway: Owing to the barrier of the Casparian strip, Cd is forced to cross the plasma membrane into endodermal cells, where its uptake depends on specific transmembrane transporters. Once inside the symplast, Cd is transferred between cells via plasmodesmata and eventually reaches the stele, where it is loaded into the xylem for long-distance translocation to the aerial parts. Because the rice plant lacks Cd-specific transporters, Cd uptake predominantly occurs through molecular mimicry by competitively utilizing transporters intended for other essential metal ions. These metal transporters include OsNRAMP1 and OsNRAMP5 from the natural resistance-associated macrophage protein (NRAMP) family [92,107], OsIRT1 and OsIRT2 from the iron-regulated transporter (IRT) family [84], OsCd1 from the major facilitator superfamily (MFS) [85], and OsZIP5 and OsZIP9 from the zinc/iron-regulated protein (ZIP) family [86].

Among these transporter proteins, OsNRAMP5 is the main transporter that mediates rice Cd absorption. This protein, located on the plasma membrane of root exodermis and distal endodermis in the mature root zone, catalyzes manganese (Mn) and Cd absorption [80]. *OsNRAMP5* knockout (KO) substantially lowers the accumulation of Cd in rice plants and grains, but simultaneously inhibits Mn uptake, leading to impaired plant growth, particularly under low Mn conditions; supplementation with high Mn concentration can partially alleviate this defect [81,82,83]. OsNRAMP1, also located on the plasma membrane, catalyzes Cd and Mn uptake; its gene expression is co-induced by iron deficiency and Cd stress. *OsNRAMP1* KO markedly declines Cd buildup in roots and aerial tissues. OsNRAMP1 and OsNRAMP5 share similar functions but are nonredundant proteins; double *OsNRAMP1* and *OsNRAMP5* mutants exhibit more severe defects in Cd and Mn uptake, indicating that OsNRAMP1 and OsNRAMP5 synergistically regulate Cd uptake [79]. Moreover, OsIRT1 and OsIRT2 coordinate with OsNRAMP1 and OsNRAMP5 to control Cd uptake [84]. OsCd1, another key transporter, is also localized on the root cell membrane and directly participates in Cd absorption by roots, promoting its grain accumulation [85]. Furthermore, OsZIP5 and OsZIP9, as zinc (Zn)/Cd influx transporters, play crucial roles in regulating Zn and Cd uptake. Their double mutants exhibit more severe Zn deficiency symptoms and substantially decreased Cd uptake capacity, further confirming that these proteins have a synergistic role in Zn/Cd absorption [86]. The RING-type E3 ubiquitin ligase OsHIR1 interacts with multiple substrate proteins and catalyzes their degradation by involving the ubiquitin-26S proteasome pathway, thereby regulating heavy metal uptake. *OsHIR1* overexpression in *Arabidopsis thaliana* enhances tolerance to Cd and reduces heavy metal accumulation in shoots and roots [87]. Finally, OsABCG43 is predominantly expressed in leaf and root vascular tissues and is extensively upregulated following Cd exposure stress. *OsABCG43* overexpression increases the accumulation of Cd in rice, which eventually suppresses photosynthesis and plant growth and increases rice plant sensitivity to Cd stress [88].

### 4.2. Foliar Uptake

Foliar uptake of environmental Cd serves as another significant source of Cd accumulation in rice. However, the precise molecular mechanisms and pathways underlying foliar Cd uptake remain incompletely understood. Atmospheric Cd is predominantly deposited as easily soluble and acid-extractable forms, which exhibit high bioavailability and easy absorption through leaves. Atmospheric Cd enters the leaf interior through the stomata; some of the absorbed Cd is sequestered and detoxified within the leaves, while the remaining portion is translocated through the phloem to grains, with limited downward transport to roots [108,109]. For example, *OsMTP11*, which is highly expressed in vascular parenchymal cells of rice leaves, interacts with the vacuolar sorting receptor OsVSR2 to mediate Cd sequestration into vacuoles through the endomembrane system, thereby reducing cellular Cd mobility and toxicity [106]. Foliar Cd uptake contributes majorly to overall plant Cd accumulation and cannot be neglected. Recent isotopic tracing and field experiments indicate that rice exposed to areas near smelters absorbs 45–70% of the total plant Cd content, contributing even more to grain Cd accumulation than root uptake [108,110,111]. Xu et al. [112] conducted a simulated Cd wet deposition experiment and found that Cd can remain on rice leaf surface for a longer duration and continue to be absorbed by the plant, resulting in an accumulation of 11.23–15.53 μg Cd/plant. Within the plant, Cd is primarily enriched in the leaves and gradually translocated to the grains; the maturity stage is crucial to facilitate Cd accumulation. This finding highlights the significant contribution of foliar absorption to Cd accumulation in rice, challenging the traditional view that root uptake is the primary pathway. The relative importance of foliar absorption may be influenced by various factors, such as the level of atmospheric deposition, growth stages, and rice’s capacity for Cd uptake. In certain regions, particularly in more polluted environments, foliar absorption may become the dominant pathway for Cd accumulation, even contributing more to grain cadmium levels during certain growth stages, such as flowering and maturation.

### 4.3. Xylem-Mediated Transport (Translocation from Roots to Shoots)

Upward Cd transport through xylem loading primarily determines Cd distribution within the rice plant; this process regulates the initial Cd influx from roots to aboveground tissues such as stems, leaves, and grains, thereby influencing the grain Cd accumulation level [113]. According to several reports, the variation in grain Cd levels among different rice cultivars mainly depends on Cd concentration and flow rate in the xylem rather than the root absorption capacity. Cd can promptly reach the panicle through the xylem, and a faster flow rate corresponds to a higher grain Cd accumulation level [113,114].

#### 4.3.1. Cell Wall Fixation and Vacuolar Compartmentalization

Following root absorption, rice plants limit Cd translocation to aboveground tissues primarily through cell wall immobilization and vacuolar sequestration to alleviate toxic effects. The root cell wall is the primary barrier preventing upward Cd transport, where Cd binds predominantly to polysaccharide components such as pectin and hemicellulose. The chemical composition and structure of the cell wall directly determine its ability to bind Cd. Exogenous application of silicon [115], nitric oxide (NO) [116], selenite [117], and brassinosteroids [118] can promote the accumulation of pectin, hemicellulose, and cellulose, thereby augmenting Cd fixation in the cell wall and considerably reducing its translocation to shoots. In addition to cell wall immobilization, vacuolar sequestration is pivotal to maintain Cd homeostasis. Multiple vacuolar membrane-localized transporters actively transport Cd into vacuoles, thereby reducing cytosolic Cd concentration and toxicity. Among these, OsHMA3, a P1B-type ATPase, mediates the cytosol-to-vacuole efflux of Cd [103]. Furthermore, *OsHMA3* overexpression in high-Cd-accumulating cultivars markedly enhances vacuolar Cd sequestration in roots, reducing grain Cd content by 94–98%, i.e., nearly to undetectable levels, without compromising yield or micronutrient accumulation [119]. Another key transporter, OsABCC9, a vacuolar membrane-localized C-type ABC transporter, facilitates Cd uptake in vacuoles, thereby lowering cytoplasmic Cd toxicity and bioavailability. Yang et al. [104] reported that, compared to the wild-type plant, *OsABCC9* KO mutants exhibit increased Cd sensitivity and remarkably high levels of Cd in shoots, roots, and grains, indicating its crucial involvement in restricting Cd translocation to shoots. OsNRAMP2, a metal transporter localized on the vacuolar membrane, primarily mediates the vacuole-to-cytoplasm efflux of Cd, promoting Cd transfer from roots to stems, leaves, and grains. *OsNRAMP2* KO reduces grain Cd content by over 38%, whereas its overexpression increases grain Cd content by more than 50%; this finding highlights its critical role in negatively regulating Cd accumulation [91]. Additionally, the vacuolar membrane protein OsCAX2 actively transports Cd into vacuoles to enable its sequestration and detoxification. *OsCAX2* overexpression enhances Cd tolerance in rice, reduces Cd root-to-shoot translocation, and lowers Cd levels in xylem sap and grains [105].

#### 4.3.2. Xylem-Mediated Transport

Before Cd enters the rice xylem for long-distance transport, it must first traverse the root exodermis, cortex, and endodermis. This radial transport is tightly regulated by cellular barriers, namely suberin lamellae and the Casparian strip, in coordination with specific transport proteins [120]. The efficiency of this process determines whether Cd can successfully enter the stele and load into the xylem to facilitate its upward translocation. In this process, OsNRAMP1, located on the plasma membranes of the root endodermis and pericycle, may play a key role in facilitating the crossing of Cd through the endodermal barrier and its subsequent loading into the xylem. [90]

During the process of Cd entering the xylem of rice, several key transporters participate in Cd transport, including members of the P-type ATPase (HMA) superfamily, cation/Ca^2+^ antiporter (CaCA) superfamily, low-affinity cation transporter (LCT) family, NRAMP family, and ZIP family. Among these transporters, OsHMA2 is currently recognized as the primary Zn/Cd xylem-loading transporter. OsHMA2, located on the plasma membrane, is primarily expressed in root and nodal vascular tissues; it directly mediates Cd efflux from stele cells into the xylem, and its loss of function markedly inhibits upward Cd translocation [89]. Similarly, OsZIP7, localized on the plasma membrane, is expressed in metaxylem parenchymal cells of roots and nodes; its KO facilitates the retention of Cd in roots, establishing its vital role in inter-nodal Cd transport and xylem loading [95]. In addition to these two key transporters, OsCCX2, a protein with high expression in the xylem region of nodes, is proposed to function as a plasma membrane Cd efflux transporter that facilitates xylem loading; its KO remarkably reduces Cd accumulation in aerial tissues and grains [96]. OsLCT2, primarily expressed in the root stele and cells near the xylem, inhibits Cd loading; moreover, its overexpression reduces Cd entry into the xylem, thereby lowering Cd buildup in grains and shoots [97]. Although OsNRAMP5 primarily mediates initial Cd uptake at the root surface, it simultaneously contributes to the transportation of Cd into the xylem [66]. OsZIP2 is also highly expressed in root xylem parenchyma and nodal vascular tissues; it modulates Cd transport between the root and stem and regulates Cd distribution among nodal vascular bundles, thereby affecting Cd redistribution throughout the plant. *OsZIP2* KO significantly enhanced the translocation of Cd from roots to stems, increased its allocation to flag leaves, but reduced its distribution to grains. In contrast, overexpression of *OsZIP2* suppressed Cd translocation to stems, thereby rendering rice more sensitive to Cd, possibly due to impaired Cd sequestration or detoxification mechanisms in roots [93]. OsCAL1, a defensin-like protein in rice, also mediates cytosol-to-apoplast Cd efflux, thereby reducing intracellular Cd concentration [98]. Collectively, these Cd transporters, primarily expressed in roots and nodes, synergistically regulate Cd entry into the xylem and form a molecular network to control distant Cd transport and final accumulation in rice grains.

### 4.4. Phloem Transport (Grain Translocation)

Following absorption by rice roots, Cd is mobilized to grains mainly through two pathways: (1) root-absorbed Cd is directly transported to grains by a xylem-mediated pathway; however, this pathway contributes little to Cd accumulation due to the weak transpiration of grains and (2) Cd is primarily transported upward through the xylem to vegetative organs such as stems and leaves and subsequently translocated into grains through the phloem during the reproductive stage, which is the main route of grain Cd accumulation. Atmospheric Cd taken up by leaves is also redistributed to grains through the phloem, although part of it is immobilized within the leaves. As shown previously, most Cd (91–100%) present in rice grains during the reproductive phase is derived from phloem transport, highlighting the dominant role of the phloem in grain Cd accumulation [121]. This process is mediated by the coordinated regulation of multiple key transporters, including OsLCT1, DEF8, and OsHMA2. OsLCT1, located on the plasma membrane, shows high expression around the phloem vascular bundles of reproductive-stage leaves and upper nodes, facilitating Cd efflux from cells into the apoplast or phloem to promote grain Cd accumulation [99]. DEF8, an extracellularly localized defensin-like protein secreted by vesicles, participates in Cd unloading in the grain phloem; its mutation significantly reduces grain Cd content without affecting nutrient accumulation or agronomic traits [100]. OsHMA2, with a high expression level in the phloem of roots and nodes, is involved in Cd loading into the xylem and preferential phloem transport to developing grains; *OsHMA2* deletion markedly inhibits the translocation of Cd from roots to grains, causing Cd buildup in roots but reduction in grains [101,102]. Thus, the phloem and its transport proteins have major functions in Cd redistribution and grain enrichment, serving as key molecular pathways for regulating Cd accumulation and ensuring food safety.

## 5. Integrated Strategies for Cd Pollution Control in Rice

**Figure 3:**
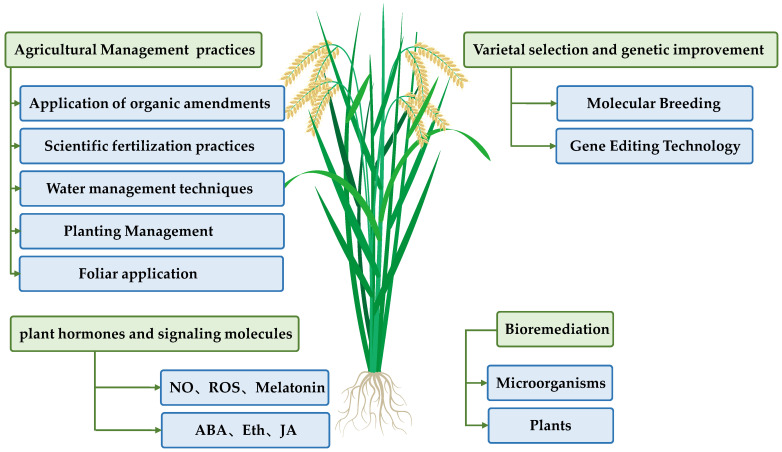
Schematic Diagram of Integrated Strategies for Cadmium Pollution Control in Rice.

To ensure adequate rice yield and food safety, experts in agriculture and environmental sciences are focusing their efforts to effectively reduce rice Cd content. Given that Cd pollution sources are complex, its migration mechanisms are diverse, and rice varieties show varying responses to Cd exposure, it is imperative to adopt multilevel and coordinated integrated control strategies, which primarily include agricultural management practices, cultivar improvement, bioremediation technologies, and phytohormone regulation. Figure 3 provides a schematic diagram of these integrated strategies for Cd pollution control in rice, while Table 2 offers a comparative analysis of the advantages, limitations, and application prospects of different Cd mitigation strategies.

### 5.1. Agricultural Management Practices

Implementing appropriate agricultural management approaches is the key to control Cd pollution in soils and crops and ensure adequate food safety and ecological health. The following common measures are implemented to contain and reduce Cd contamination. Table 3 summarizes representative approaches for these strategies, along with their mechanisms and potential applications.

(1) Application of organic amendments, including biochar [134], compost [56], and crop residues [133,149], and inorganic amendments such as lime [135], zeolite [136], and bentonite [137]. These amendments minimize the bioavailability of Cd in soil through mechanisms such as adsorption, complexation, and transformation, effectively decreasing Cd uptake and accumulation in rice and other crops. According to Liu et al. [138], liming decreased bioavailable Cd content in soil and Cd concentration in aboveground plant tissues by 19.2–29.4% and 29.3–36.3%, respectively. However, the long-term effects of these ameliorants and the changes in soil microbial communities still require further evaluation. Additionally, the application rate and effectiveness of ameliorants are influenced by various factors such as soil type and environmental conditions, and therefore need to be adjusted and optimized based on specific circumstances.
(2) Targeted fertilization practices also contribute remarkably to mitigate Cd pollution. Rational application of fertilizers such as urea [139], phosphate [140], sulfur [141], selenium [142], and silicon [143] improves soil conditions, enhances crop stress resistance, and reduces Cd accumulation. Zhou et al. [150] observed that applying urea in the ratio of 30% at tillering, 40% at panicle initiation, and 30% at heading lowered Cd content in brown rice by 40.7% compared to that achieved by conventional fertilization.
(3) Water management techniques, including intermittent irrigation, continuous flooding, and alternate wetting and drying, modulate soil redox status, promote the formation of iron plaques, and inhibit Cd activation and mobility, thereby reducing plant Cd uptake [32,144,151,152]. However, these water management practices may be limited by water availability and cost in arid or water-scarce regions. Furthermore, the adaptability of water management varies across different regions and needs to be flexibly adjusted based on local water resource conditions.
(4) Crop rotation and fallowing effectively reduce the bioavailability of Cd in soil. As reported previously, following 2–3 years of rotation, Cd levels in brown rice decreased to below safety thresholds, with reduction levels ranging from 37% to 73% [145,146]. Although crop rotation can effectively reduce the bioavailability of cadmium in the soil, its implementation is still limited by factors such as arable land area, crop variety selection, and climate conditions. In some regions, the lack of suitable alternative crops or the long growing cycle of rice may limit the effectiveness of crop rotation.
(5) Foliar application of metal chelates (e.g., iron chelates), nonmetallic compounds, or organic foliar agents is a novel approach to alleviate Cd-induced toxicity. These treatments enhance chlorophyll content, improve photosynthesis, enrich antioxidant defense systems, and decrease cell membrane permeability, ultimately increasing plant tolerance to heavy metals [147,148,153,154]. For instance, Wang et al. [147] demonstrated that spraying 50 mg/L iron chelate reduced the content of Cd in brown rice by 29% and increased the enzymatic activities of POD and SOD by 54.4% and 51.6%, respectively, facilitating ROS scavenging and decreasing oxidative stress. However, the application of foliar ameliorants often requires multiple sprays, which increases production costs. Moreover, the effectiveness of ameliorants varies significantly across different rice varieties and other crops, and frequent use may also lead to secondary environmental pollution.

### 5.2. Varietal Selection and Genetic Improvement

Varietal selection and genetic improvement represent a highly effective and sustainable strategy to mitigate Cd contamination in rice. Rice cultivars exhibit remarkable differences in their capacity to accumulate Cd in grains; thus, screening and promoting low-Cd-accumulating varieties can substantially reduce grain Cd levels without altering cultivation practices [155,156]. Recent advances in molecular breeding and gene editing technologies, such as CRISPR/Cas9, provide robust tools for precisely regulating Cd uptake and transport. Targeted modulation of key Cd transporter genes—including *OsNRAMP5*, *OsNRAMP1*, and *OsHMA3*—can effectively reduce Cd absorption and translocation to grains without adverse effects on yield or quality. For example, *OsNRAMP5* KO in brown rice reduces Cd content by 97.8–98.3%, while *OsNRAMP1* knockdown decreases Cd content in roots and shoots by 23.1–28.3% and 34.0–39.7%, respectively [79,157]. Nevertheless, potential yield penalties or micronutrient deficiencies (e.g., reduced Mn uptake observed in *OsNRAMP5* KO) remain important challenges that need to be addressed alongside Cd reduction. However, despite the tremendous potential of gene editing technology in cadmium pollution control in rice, regulatory barriers for genetically modified crops remain a significant challenge in advancing the application of these technologies. There are differences in regulatory policies for genetically modified crops across countries and regions, with some countries still adopting a cautious approach toward genetic modification, which may limit the promotion of related technologies. Additionally, molecular marker-assisted selection and genome-wide association studies have been widely utilized to identify and introgress low-Cd alleles, accelerating low-Cd cultivar breeding [158,159]. Combining resources that promote natural variation with mutagenesis approaches such as ion-beam irradiation also facilitates the development of genetically stable low-Cd mutants [160,161]. Overall, varietal selection and genetic improvement, particularly in combination with molecular biology techniques, not only control Cd pollution in rice at the source level but also provide a robust foundation for ensuring rice safety and sustainable agricultural development.

### 5.3. Regulation of Cd Tolerance Through Plant Hormones and Signaling Molecules

Following Cd stress exposure, various signaling molecules (e.g., NO, ROS, and melatonin) [162,163,164] and hormones (including ethylene, jasmonic acid, and abscisic acid) [165,166,167] in rice exhibit dynamic changes in their levels and play critical roles in regulating antioxidant defenses, osmotic adjustment, and ion homeostasis mechanisms for Cd tolerance. These molecules enhance the tolerance of rice plants to Cd stress by modulating the expression of antioxidant systems, osmolytes, and metal transporter genes. Exogenous application of hormones such as auxin (indole acetic acid) and its precursor L-tryptophan promotes rice growth and yield, while increasing Cd immobilization in roots, thereby reducing Cd translocation to grains and lowering Cd content in rice [168,169]. Melatonin, as a signaling molecule, substantially decreases Cd accumulation in rice leaves when applied as foliar spray, improves photosynthetic efficiency, enhances antioxidant enzyme activities, and regulates the expression of Cd transport-related genes to limit Cd translocation within the plant [170,171,172]. Liu et al. [173] demonstrated that foliar melatonin application reduced Cd content by 19–38% in rice leaves and grains, effectively inhibiting Cd accumulation and translocation to grains. Overall, by scientifically applying plant hormones and signaling molecules, it is possible to alleviate cadmium toxicity, promote rice growth, and increase yield, while effectively reducing cadmium accumulation in rice. This provides a feasible solution for the green control of cadmium contamination in rice. In the future, combining hormone regulation with molecular breeding techniques holds promise for developing rice varieties with high cadmium tolerance and low cadmium accumulation, thereby ensuring safe production. Further understanding the mechanisms of action of these signaling molecules will not only optimize hormone application strategies but also provide new regulatory targets for rice breeding, enhancing cadmium tolerance and ensuring food security and sustainable development.

### 5.4. Bioremediation

Bioremediation of Cd pollution in rice primarily depends on the synergistic interactions between microorganisms and plants to reduce Cd bioavailability in soil or promote its immobilization, thereby decreasing Cd absorption and buildup in rice. Recent studies have identified various Cd-tolerant or Cd-accumulating microorganisms such as *Bacillus vietnamensis*, *Pseudomonas aeruginosa*, and *Bacillus subtilis*, which can effectively reduce soil Cd bioavailability through adsorption, precipitation, complexation, or redox reactions; this leads to a reduced Cd content in rice while simultaneously promoting plant growth and stress resistance [129,174,175,176]. Moreover, the combined application of microorganisms with remediation materials such as biochar enhances rice growth parameters, photosynthetic performance, chlorophyll content, and antioxidant enzyme activities, further mitigating Cd accumulation in rice [177]. Concurrently, phytoremediation serves as a crucial approach to reduce soil Cd levels by cultivating high-biomass, fast-growing plants with strong capabilities to accumulate heavy metals (e.g., *Taraxacum officinale*, *Solanum nigrum*, *Cyperus* spp., and *Myriophyllum* spp.), which transfer and sequester Cd in their aboveground tissues. Periodic harvesting removes the accumulated Cd from the ecosystem, thus lowering Cd load in the environment [178,179,180,181,182]. In addition, some plants (e.g., *Cyperus malaccensis*, *Phragmites australis*) are typical silicon accumulators that can enrich large amounts of Si and form phytoliths [183]. Phytoliths may act as “micro-mineralization warehouses” for Cd, enabling its long-term sequestration and reducing the risk of Cd transfer through the food chain. In recent years, the long-term immobilization of Cd in phytoliths has emerged as a potential new strategy for the phytoremediation of Cd-contaminated soils [184,185]. Nevertheless, the drawback of phytoremediation lies in its long remediation time, often requiring prolonged plant growth and multiple harvests to effectively reduce cadmium levels in the soil. This makes the phytoremediation process slow, potentially unable to rapidly address soils with severe heavy metal contamination. Additionally, the effectiveness of phytoremediation is closely related to plant species, soil types, and environmental conditions, which may lead to variability in results. Therefore, despite being an environmentally friendly, low-cost, and flexible method, phytoremediation technology still needs to be combined with other approaches, such as molecular breeding and agricultural management, to build a multi-dimensional and multilevel integrated remediation system for cadmium pollution in rice, ensuring food security and promoting soil health more effectively.

## 6. Future Perspectives

Rice Cd pollution not only poses a risk to food security and human health, but it is also a major challenge to the sustainable development of agricultural ecosystems. Therefore, in-depth research on mechanisms associated with Cd pollution in rice and its control technologies are theoretically significant and practically relevant. Currently, although various agricultural management, bioremediation, and molecular breeding strategies have made positive progress, the accumulation of Cd in rice is regulated by multiple factors, including genetics, environment, and cultivation conditions. There is an urgent need to strengthen interdisciplinary collaboration and concerted efforts. In this context, understanding the molecular mechanisms of Cd uptake, translocation, and accumulation in rice is of critical practical importance for formulating targeted mitigation strategies. It is particularly noteworthy that this review is the first to clearly summarize the role of foliar absorption as one of the pathways for cadmium uptake in rice. Traditionally, cadmium accumulation in rice has primarily been focused on root uptake and translocation; however, foliar absorption plays a significant role that should not be overlooked, especially when atmospheric cadmium concentrations are high. Studies have shown that rice not only absorbs cadmium through the roots but may also directly absorb pollutants from the atmosphere through the leaves. Understanding the mechanisms of foliar absorption provides a new perspective for monitoring and managing cadmium pollution. This finding not only helps optimize rice planting practices to reduce cadmium accumulation but also offers potential regulatory measures for controlling atmospheric emissions around paddy fields. Moreover, functional studies of key genes involved in Cd uptake and translocation in rice, such as *OsNRAMP1*, *OsHMA3*, and *OsNRAMP5*, provide clear targets for precision breeding and gene editing. By regulating the expression or function of these genes, it is possible to effectively reduce cadmium absorption in rice and inhibit its translocation to the grains, thereby enhancing rice safety. For example, *OsNRAMP5* KO can reduce cadmium accumulation in rice by 97.8–98.3% without affecting yield, providing a breeding direction for low-cadmium rice varieties. Future studies should further integrate advances from molecular biology, soil science, ecology, and agricultural engineering to improve translational efficiency from laboratory research to field applications. Additionally, accelerating the exploration and innovation of low-Cd rice germplasm resources and constructing a diverse, stable, and efficient breeding material repository will provide a solid foundation for developing safe and high-quality rice cultivars, thereby contributing greatly to agricultural green transformation and human safety.

## Figures and Tables

**Table 1:** Major Proteins Involved in Cd Uptake, Movement, and Detoxification in Rice.

Proteins	Family/Protein Product	Normal Substrates (Physiological Role)	Transportation	References
**Root Uptake**				
OsNRAMP1	Natural resistance-associated macrophage protein	Mn^2+^, Fe^2+^	Mediates Cd uptake from soil into root cells	[79]
OsNRAMP5	Natural resistance-associated macrophage protein	Mn^2+^, Fe^2+^	Facilitates Cd uptake into rice roots from soil	[80,81,82,83]
OsIRT1	Iron-regulated metal transporter; Fe(II) transporter gene	Zn^2+^, Fe^2+^	Mediates Cd influx into root cells from soil	[84]
OsIRT2	Iron-regulated metal transporter; Fe(II) transporter gene	Fe^2+^	Mediates Cd uptake from soil into root cells	[84]
OsCd1	Major facilitator superfamily domain-containing protein	Unknown	Mediates Cd absorption in roots	[85]
OsZIP5	ZRT- and IRT-like protein; zinc transporter	Zn^2+^	Mediates Cd uptake from soil solution into root cells	[86]
OsZIP9	ZRT- and IRT-like protein; zinc transporter	Zn^2+^	Mediates Cd uptake from soil into rice root cells	[86]
OsHIR1	RING finger protein; heavy metal induced RING E3 ubiquitin ligase 1	Not a transporter	Enhances Cd uptake into roots through modulation of transporter activity	[87]
OsABCG43	ATP binding cassette (ABC)-type transporter	Unknown	Mediates Cd influx into roots from environment or xylem	[88]
**Xylem-Mediated Transport**				
OsHMA2	Heavy metal ATPase 2	Zn^2+^	Facilitates root-to-shoot Cd transport through xylem	[89]
OsNRAMP1	Natural resistance-associated macrophage protein	Mn^2+^, Fe^2+^	Facilitates Cd loading into xylem for root-to-shoot translocation	[90]
OsNRAMP2	Natural resistance-associated macrophage protein	Fe^2+^	Mediates vacuolar Cd efflux and facilitates Cd translocation	[91]
OsNRAMP5	Natural resistance-associated macrophage protein	Mn^2+^, Fe^2+^	Affects Cd translocation via xylem	[92]
OsZIP2	ZRT- and IRT-like protein; zinc transporter	Zn^2+^, Mn^2+^	Transports root-absorbed Cd to aboveground tissues via the xylem	[93]
OsZIP3	ZRT- and IRT-like protein; zinc transporter	Zn^2+^	Mediates Cd translocation from roots to shoots in rice	[94]
OsZIP7	ZRT- and IRT-like protein; zinc transporter	Zn^2+^, Fe^2+^	Promotes Cd xylem loading and inter-node transfer to grains	[95]
OsCCX2	Na^+^/Ca^2+^ exchanger	Ca^2+^, Na^+^, Fe^2+^	Enhances Cd transport to grains via xylem loading	[96]
OsLCT2	Low-affinity Cation Transporter 2	Unknown	Limits Cd xylem loading and root-to-shoot transport	[97]
OsCAL1	Cell wall localized defensin protein	Unknown	Mediates Cd efflux to the apoplast	[98]
**Phloem Transport**				
OsLCT1	Low-affinity Cation Transporter 1	K^+^, Mg^2+^, Ca^2+^, Mn^2+^	Promotes Cd phloem loading and transport to grains	[99]
DEF8	Defensin-like protein 8	Not a transporter	Facilitates Cd unloading from the phloem into grains	[100]
OsHMA2	Heavy metal ATPase 2	Zn^2+^	Promotes the phloem loading and transport of Cd to developing grains	[101,102]
**Vacuolar Cd Compartmentalization**				
OsHMA3	Heavy metal ATPase 3	Zn^2+^	Sequesters Cd into vacuoles to restrict its translocation to shoots	[103]
OsABCC9	C-type ABC transporter	GSH, PC–Metal Complexes	Limits the transport from roots to stems	[104]
OsCAX2	Cation/H^+^ Exchanger	Ca^2+^	Limits the transport from roots to stems	[105]
OsMTP11	Metal tolerance protein	Mn^2+^	Promotes vacuolar Cd sequestration in leaf vasculature, limiting transfer to grains	[106]

**Table 2:** Comparative analysis of Cd mitigation strategies in rice.

Type of Strategy	Main Mechanisms and Advantages	Limitations	Representative Measures/Progress	References
Agricultural management	Regulating soil pH, organic matter, moisture, and nutrients to reduce Cd bioavailability.	Effect influenced by soil type and climate, requiring continuous input.	Lime/biochar/organic fertilizer, crop rotation, and irrigation management.	[17,28,122,123,124]
Varietal improvement	Breeding or genetically modifying low-Cd-accumulating varieties to block Cd entry into grains at the source.	Long breeding cycle with the need to balance adaptability and yield.	Molecular breeding with *OsHMA3*/*OsNRAMP5* and QTL pyramiding.	[125,126,127]
Plant hormones/signaling molecules	Enhancing antioxidant capacity and regulating transporters to alleviate Cd toxicity.	Mostly short-term exogenous treatments with limited field application.	Exogenous melatonin, brassinosteroids, NO, etc.	[28,124,128]
Bioremediation	Adsorbing/immobilizing Cd to reduce its bioavailability in soil.	Long remediation cycle with limited economic and field feasibility.	Intercropping with hyperaccumulator plants and inoculation with Cd-adsorbing bacteria.	[17,129,130,131,132]

**Table 3:** Remediation effects of agricultural management practices on cadmium in paddy soils.

Strategies	Treatment	Results	References
**Soil amendment**			
Organic amendments	Biochar and vermicompost (5 t/hm^2^ biochar + 5 t/hm^2^ vermicompost)	Cd (plant concentration) was significantly reduced by 72%	[56]
	Biochar (adsorption)	Cd^2+^ adsorption capacity was maximum at 244.43 mg/g	[133]
	Biochar (4%)	Cd (soil solution) was significantly reduced by 67%	[134]
Inorganic amendments	Moisture and lime (2% lime)	Suppress bioavailability and toxicity of soil Cd	[135]
	zeolite@cellulose-poly (acrylamide) hydrogel (2.5 *w*/*w*)	The bioavailable and total Cd concentrations were reduced by 59.38% and 1.75%, respectively	[136]
	FeMg-LDH/Bentonite to compost (3:7, 1:1, 7:3)	Reduce soil cadmium bioavailability and plant cadmium concentration.	[137]
	Lime (CdL5, CdL15 and CdL20)	Reduced soil available Cd by 19.2–29.4% and shoot Cd by 29.3–36.3%	[138]
**Scientific fertilization practices**			
Compound fertilizer	Compound fertilizer (600 kg/ha)	Cd (grain Cd content) decreased by 26.46–56.53%	[139]
Phosphate	Phosphate (0, 0.05 and 0.5 mmol/L)	Boost antioxidant defense and reduce Cd toxicity in soybeans	[140]
Na_2_SO_4_	Na_2_SO_4_ (2.64 and 5.28 mM)	Cd (grain Cd content) decreased by 23.5% and 39.5%, respectively	[141]
Selenium fertilizer	Soil Se content (0.25, 0.375, 0.50, 0.75 and 1.00 mg·kg^−1^)	Cd (grain Cd content) was reduced by 48.4%~82.89%	[142]
silicon and nitric oxide	Si and NO (100 μM Sodium nitroprusside + 3293.3 kg/ha K2SiO3)	Cd (grain Cd content) decreased by 66%	[143]
**Water management techniques**			
Intermittent irrigation	Three-day flooding and five-day drainage	Significant reduction in grain Cd content.	[32]
Long-term flooding	A 3–5 cm deep water layer maintained on the soil surface until rice harvest	Cd content in rice husk and grains was significantly reduced by 90.2%	[144]
**Crop rotation and fallowing**			
Rotation	An oilseed rape-rice rotation	In the second year, the minimum grain Cd concentrations of the two varieties were 0.10 and 0.11 mg kg^−1^, respectively	[145]
	A rice–chicory rotation	Effectively reduce cadmium accumulation in subsequent rice crops	[146]
**Foliar ameliorant**			
Foliar iron fertilization	Foliar iron fertilization (20, 50 and 100 mg/L)	The 50 mg/L treatment reduced grain Cd concentration by 29.0%	[147]
Foliar application fulvic acid	fulvic acid (0.5 g/L)	Significantly alleviated Cd-induced toxicity symptoms in lettuce	[148]

## Data Availability

The data will be provided by the authors upon reasonable request.

## Abbreviations

The following abbreviations are used in this manuscript:

Cd	Cadmium
PSII	Photosystem II
Rubisco	Ribulose-1,5-bisphosphate carboxylase/oxygenase
ROS	Reactive oxygen species
O_2_^−^	Superoxide anions
H_2_O_2_	Hydrogen peroxide
POD	Peroxidase
SOD	Superoxide dismutase
KO	Knockout
NO	Nitric oxide
